# Chitosan‐Coated Rainbow Trout Fillets With *Chlorella vulgaris* Hydrolysate: Shelf‐Life Extension Under Refrigeration

**DOI:** 10.1002/fsn3.70378

**Published:** 2025-06-05

**Authors:** Nadia Yazdani, Sakineh Yeganeh, Mina Esmaeili Kharyeki, Shahab Naghdi

**Affiliations:** ^1^ Department of Fisheries, Faculty of Animal Science and Fisheries Sari Agricultural Sciences and Natural Resources University Sari Iran; ^2^ Seafood Processing Department, Marine Sciences Faculty Tarbiat Modares University Noor Iran

**Keywords:** antioxidant activity, *Chlorella vulgaris* hydrolysate, edible coating, natural preservatives, rainbow trout, shelf‐life extension

## Abstract

Chitosan coatings enriched with *Chlorella vulgaris* hydrolysates were evaluated for their effectiveness in preserving rainbow trout ( *Oncorhynchus mykiss* ) fillets during refrigerated storage at 4°C ± 1°C. Alcalase‐hydrolyzed *C. vulgaris* exhibited potent antioxidant activity (IC_50_: 0.30 mg/mL; IC_80_: 0.61 mg/mL). These concentrations were incorporated into chitosan coatings (designated as T3 and T4) and compared to chitosan‐coated (T2) and uncoated (T1) control fillets. TVBN values after storage were 47.87 (uncoated control), 43.02 (chitosan‐only), 36.45 (IC_50_), and 30.61 mg/100 g (IC_80_) (*p* > 0.05). Initial pH values across all treatments showed no significant differences (*p* < 0.05). Thiobarbituric acid (TBARS) values exhibited a significant increasing trend throughout the storage period (*p* > 0.05), and at the end of storage, the values were lower in all coated groups, including 0.99 mg MDA/kg (T2), 0.90 mg MDA/kg (T3), and 0.86 mg MDA/kg (T4), compared to 1.5 mg MDA/kg in the T1 group. PV peaked on Day 12 at 2.23 (control), 1.86 (chitosan‐only), 1.46 (IC_50_), and 1.00 mEq/kg (IC_80_) (*p* > 0.05). Lightness (L*) values showed a modest but significant increase during storage (*p* < 0.05). Although mesophilic and psychrotrophic bacterial counts rose in all samples, they remained lower in the coated groups (*p* > 0.05). Hydrolysate‐enriched coatings significantly delayed sensory deterioration compared to controls. Thus, chitosan coatings with *C. vulgaris* hydrolysate are recommended as effective antibacterial and antioxidant packaging solutions.

## Graphical abstract

Chitosan coatings enriched with *Chlorella vulgaris* hydrolysates (especially at IC_80_) significantly improved rainbow trout fillet preservation during refrigerated storage, reducing TVBN (30.61 vs. 47.87 mg/100 g in control), TBARS (0.86 vs. 1.5 mg MDA/kg), and PV (1.00 vs. 2.23 mEq/kg) while delaying microbial growth and sensory deterioration. The hydrolysate‐enhanced coatings outperformed chitosan‐only and uncoated controls, demonstrating potent antioxidant and antibacterial effects.

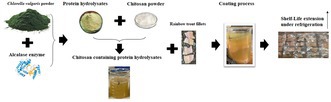

## 1 Introduction

Marine resources provide essential nutrients, including high‐quality protein and omega‐3s, critical for global nutrition (Naghdi et al. 2024). Over the last decade, with the increasing global population and demand for food resources, the importance of aquatic resources in ensuring food security and proper nutrition has become more apparent (Tsegay et al. 2024). Rainbow trout ( *Oncorhynchus mykiss* ) is a favored species in Iran and many countries worldwide, prized for its delicious taste and meat quality. Its production rate has been increasing significantly during the few past years (Taghizadeh Andevari et al. 2019). According to an FAO report, global rainbow trout production increased from 6,546,500 tons in 2015 to 739,500 tons in 2020, demonstrating its widespread consumer appeal (FAO 2022). In line with this, Iran's production of rainbow trout reached 580,000 tons in 2022 (Iran Fisheries Organization 2022). However, fresh seafood has a limited shelf life, so it is considered a perishable food owing to the fast enzymatic and bacterial spoilage that occurs after death (Kumar et al. 2023; Sáez et al. 2021; Shah Hosseini et al. 2021; Soltani et al. 2023). Seafood is highly susceptible to lipid oxidation and quality deterioration due to the high levels of unsaturated fatty acids and concentration of pigments and metal ions in its flesh (Naghdi et al. 2021; Sáez et al. 2021; Safari et al. 2018). Lipid oxidation products (e.g., hydroperoxides, aldehydes) degrade protein quality, causing off‐flavors and reduced edibility (Farsani et al. 2018; Kumar et al. 2023; Naghdi et al. 2021). Therefore, to increase the shelf life of seafood, various packaging methods, such as films and coats, are employed (Naghdi, Lorenzo, et al. 2023; Shah Hosseini 2023). Edible coatings derived from natural polymers (e.g., chitosan, starch, gelatin) have gained prominence as sustainable packaging alternatives. Their ability to enhance barrier properties, suppress microbial growth, and extend shelf life surpasses conventional materials (Mehdizadeh et al. 2020; Naghdi, Lorenzo, et al. 2023). Although chitosan‐based coatings are widely studied for seafood preservation, their limited intrinsic antioxidant activity restricts their ability to fully mitigate lipid oxidation, a critical factor in fish spoilage (Abd El‐Hack et al. 2020; Abdel‐Naeem et al. 2021; Muñoz‐Tebar et al. 2023). To address this limitation, researchers have investigated incorporating extracts from algae and microalgae with known antioxidant properties into chitosan coatings (Bagheri et al. 2016; Javadian et al. 2017; Mondal et al. 2022; Shafiei and Mostaghim 2022; Shah Hosseini 2023).

This strategy is increasingly adopted in food preservation research. Seaweeds are a valuable resource in the food and pharmaceutical industries because of their strong antioxidant effects, attributed to bioactive compounds, such as polysaccharides, minerals, vitamins, proteins, lipids, and polyphenols (Diaz et al. 2023; Dini 2023; Ijaola et al. 2024). Although algae extracts (e.g., *Padina australis*, *Palmaria palmata* ) have been integrated into coatings to enhance functionality (Harnedy and FitzGerald 2013; Soltani et al. 2023), microalgae hydrolysate offers distinct advantages: (1) its high protein content (40%–60% dry weight) yields bioactive peptides with superior free‐radical scavenging capacity (Cermeño et al. 2019); (2) its enzymatic hydrolysates exhibit broader antimicrobial activity (Sadeghi et al. 2018); and (3) its scalability (global production: 2000 tons/year) supports industrial adoption (Al‐Hammadi and Güngörmüşler 2024).

For instance, extracts from *C. vulgaris* have shown promise in delaying lipid oxidation in sardine fish fillets and inhibiting the growth of Gram‐positive lactic acid bacteria (Khanzadi et al. 2021). However, no prior work has evaluated *C. vulgaris* hydrolysate–chitosan synergies for trout preservation. This study bridges that gap by systematically evaluating how hydrolysate concentration (IC_50_ vs. IC_80_) impacts shelf‐life extension under refrigeration, addressing both oxidative and microbial spoilage pathways.

## 2 Materials and Methods

### 2.1 Chemicals and Reagents

Chemicals and reagents. Gallic acid, methanol, DPPH, medium molecular weight chitosan, and TBA were purchased from Sigma‐Aldrich. Acetic acid, glycerol, sulfuric acid, hydrochloric acid, boric acid, methyl red indicator, sodium hydroxide, and 1‐butanol were obtained from Merck. All other chemicals used were purchased from reputable suppliers and were of analytical grade.

### 2.2 Sample Collection and Preparation

*Chlorella vulgaris* powder used in this study was purchased from Caspian Company (Iran).

### 2.3 Enzymatic Hydrolysis of *Chlorella vulgaris*

The enzymatic hydrolysis was performed according to the method described by Pekkoh et al. (2021). Briefly, 2 g of *C. vulgaris* powder was mixed with 50 mL distilled water, incubated at 50°C (pH 8), and hydrolyzed with alcalase (0.1%–0.8% w/v) for 8 h. To stop the reaction, the mixture was heated to 95°C for 10 min and then quickly cooled in an ice bath. The hydrolysate was centrifuged at 5000 *g* for 10 min at 4°C, followed by filtration through Whatman filter paper No. 4. The pH of the resulting supernatant was adjusted to 7 using 1 M HCl or NaOH. The final supernatant was freeze‐dried and stored at −20°C for future analysis. For the control sample, water extraction of the algae powder was conducted by mixing 20 g of dried algae with 200 mL of distilled water in a 250 mL Erlenmeyer flask, kept in the dark, and filtered using Whatman filter paper No. 1. The filtrate was concentrated using a rotary evaporator under vacuum at 50°C (Srivastava et al. 2010). Both the hydrolysate and the control sample were analyzed for the degree of hydrolysis, soluble protein content, and DPPH radical scavenging activity. The hydrolysate with the strongest DPPH scavenging activity (lowest IC~50~/IC~80~) was selected for coatings.

### 2.4 Soluble Protein

Soluble protein content was quantified using the Lowry method (Lowry et al. 1951), employing bovine serum albumin as a standard. Absorbance readings at 570 nm were taken using a microplate reader.

### 2.5 Calculation of Hydrolysis Degree (DH)

The degree of hydrolysis was assessed following the method outlined by Naghdi, Rezaei, et al. (2023) with trichloroacetic acid. A solution was created by combining 500 μL of the hydrolyzed algal sample with 500 μL of 20% trichloroacetic acid, which was then centrifuged at 8000 *g* for 10 min. The protein concentration in the supernatant was measured using the Lowry technique, and the degree of hydrolysis was computed using this formula:

$$\begin{array}{cc} \text{Degree of hydrolysis}\;\left(\%\right)=\; & (\text{Total protein soluble in trichloroacetic acid}/\\ & \text{Protein content of the sample})\times 100 \end{array}$$

### 2.6 DPPH Radical Scavenging Activity

The DPPH radical scavenging activity was determined using a method adapted from Soltani et al. (2023). Equal volumes of sample solution (in methanol) and 1.0 mM DPPH solution (in methanol) were mixed, shaken vigorously, and incubated in the dark for 30–60 min. Absorbance was then measured at 517 nm using a UV–Vis spectrophotometer. The percentage of DPPH radical scavenging activity was calculated as follows:

$$\text{DPPH inhibition}\;\left(\%\right)=\left[\left(A_{\text{control}}-A_{\text{sample}}\right)/A_{\text{control}}\;\right]\times 100$$

where *A*_control_ = absorbance of the control (DPPH solution without sample). *A*_sample_ = absorbance of the sample (corrected for any inherent absorbance of the sample at 517 nm). The IC_50_ and IC_80_ values (concentrations required to inhibit 50% and 80% of DPPH radicals, respectively) were determined from the dose–response curve. These values were used to assess the antioxidant potential of the hydrolyzed algae.

### 2.7 Preparation of Chitosan Coatings for *Oncorhynchus mykiss* Fillets

A 2% (w/v) chitosan solution was prepared by dissolving chitosan (average molecular weight 190–310 kDa, 85% deacetylation degree) in distilled water using a heated stirrer, according to Chamanara et al. (2012). To facilitate dissolution and prevent clumping, 1% (v/v) acetic acid was added. Glycerol (0.75 mL per gram of chitosan) was incorporated as a plasticizer. The mixture was stirred continuously at 55°C. Hydrolyzed *C. vulgaris* extract, at concentrations corresponding to the previously determined IC_50_ and IC_80_ values (from the DPPH assay), was then added to separate aliquots of the chitosan solution. These mixtures were stirred continuously for 20 min (da Rocha et al. 2018).

The following treatments were prepared:

– Control (T1): *O. mykiss* without any coating.
– Chitosan 2% (T2): *O. mykiss* coated with a 2% chitosan solution.
– Chitosan 2% containing IC_50_ of microalgae hydrolysate (T3): *O. mykiss* coated with a 2% chitosan solution containing hydrolyzed algae at the IC_50_ concentration.
– Chitosan 2% containing IC_80_ of microalgae hydrolysate (T4): *O. mykiss* coated with a 2% chitosan solution containing hydrolyzed algae at the IC_80_ concentration.

The IC_50_ and IC_80_ values refer to the concentrations of hydrolyzed algae extract required to achieve 50% and 80% inhibition of DPPH radicals, respectively, as determined in the previous DPPH assay.

### 2.8 Coating Process of Fish Fillets

Live rainbow trout (approximately 500 g each) were humanely euthanized and immediately transported on ice (1:3 fish‐to‐ice ratio) to the lab. After cleaning and filleting (resulting in approximately 50 g fillets, 3–4 cm), the fillets were coated using a published method (Chamanara et al. 2012). This involved two cycles of 2‐min immersion in the coating solution followed by 30 s of air drying. After a final hour of drying, the coated fillets were refrigerated (4°C ± 1°C) for 16 days, with measurements taken every 4 days.

### 2.9 Chemical Assessments of Coated Fillets During Cold Storage

#### 2.9.1 pH and TVB (Total Volatile Base Nitrogen) of Fish Fillet During Storage

Five grams of fish fillet samples were blended with 54 mL of distilled water and then filtered through filter paper. The pH was determined at room temperature using a pH meter (Navarro‐Garcıa et al. 2004). To quantify the total volatile basic nitrogen (TVB‐N), 10 g of minced fish were placed in a distillation flask with approximately 2 g of magnesium oxide, 500 mL of distilled water, and boiling stones. Distillation was then carried out, and the resulting vapors were directed into a 2% boric acid solution that contained a few drops of indicators (methyl red and bromothymol blue). The final step involved titrating the solution with 0.1 N sulfuric acid to determine the amount of volatile nitrogen using the following equation (Ojagh et al. 2010):

$$\mathrm{mg}\;\mathrm{TVB}-N=\left(\text{Concentration of acid}\times \text{Volume of acid}\times 14\times 100\right)/10$$

#### 2.9.2 Evaluation of Lipid Oxidation of Fish Fillets

##### 2.9.2.1 Thiobarbituric Acid (TBA) Analysis

For TBA measurement, 200 mg of the fish sample was added to a 50 mL volumetric flask and diluted with butanol. Five milliliter of this dilution were then placed in a sealed tube, and 5 mL of thiobarbituric acid reagent were introduced. The tubes were incubated in a water bath at 59°C for 2 h. After cooling to room temperature, the absorbance was recorded at 530 nm using a spectrophotometer, with distilled water serving as a blank. The TBA value was calculated using the following formula (Ojagh et al. 2010):

$$\begin{array}{cc} \mathrm{TBA}=\; & \left(200/50\right)\times (\text{Absorbance of distilled water}\;\mathrm{at}\;530\;\mathrm{nm}-\\ & \text{Absorbance of sample}\;\mathrm{at}\;530\;\mathrm{nm}) \end{array}$$

##### 2.9.2.2 Peroxide Value (PV) Analysis

To determine the peroxide value, the samples were first homogenized. A total of 51 g of the homogenized fish meat was combined with 60 mL of methanol and 60 mL of chloroform. After 42 h, 36 mL of distilled water was added to facilitate phase separation. Following an additional 2 h, the extracted oil was isolated. Next, 250 mL of this fish oil was measured into an Erlenmeyer flask, and approximately 52 mL of a 3:2 chloroform‐acetic acid solution was introduced. Subsequently, 0.5 mL of saturated potassium iodide solution, 30 mL of distilled water, and 0.5 mL of 1% starch solution were added. The released iodine was then titrated with 0.01 N sodium thiosulfate solution. The peroxide value was calculated using the formula below (Hathwar et al. 2011):

$$\begin{array}{cc} \mathrm{PV}=\; & \left(\text{Oil weight}/1000\right)\times \left(\text{Thiosulfate normality}\right)\times \\ & \left(\text{Titrant volume consumed}\right) \end{array}$$

### 2.10 Microbial Tests on Fish Samples

#### 2.10.1 Total Bacterial Count

Fish muscle tissue (5 g) was aseptically collected from the sterile inner portion and homogenized in 45 mL of physiological saline solution. Serial dilutions were then created. One milliliter of each dilution was plated onto plate count agar (PCA) plates. The plates were incubated at 37°C for 41 h to assess the total bacterial load. After incubation, colonies were counted, log‐transformed, and reported as log CFU/g (Song et al. 2012).

#### 2.10.2 Psychrophilic Bacterial Count

In a sterile environment using a laminar flow hood, 5 g of fish fillet was aseptically collected with sterile forceps and scissors, placed in a sterile plastic bag, and combined with 45 mL of sterile distilled water. The bag was homogenized using a stomacher for 1 min. Serial dilutions were prepared and spread‐plated onto nutrient agar plates. The plates were incubated at 37°C for 84 h to count mesophilic aerobic bacteria, and at 10°C for 7–10 days to enumerate psychrophilic bacteria (Uçak et al. 2011).

### 2.11 Sensory Evaluation of Fillets

Sensory qualities of rainbow trout fillets, including color, odor, texture, taste, and overall acceptability, were evaluated by a 15‐member semi‐trained panel with an average age of 25 years throughout storage. Panelists participated in regular sessions three times a week for 4 weeks to familiarize themselves with the sensory characteristics of raw fish, including undesirable odor, texture, and color. It is noteworthy that the evaluators were students in the Fisheries group and had some prior familiarity with fish quality assessment before the training. Fresh fillets, frozen at −20°C and thawed for 3 h before assessment, served as the gold standard. Fried samples were scored on a 0–10 scale (10 being highest quality, and scores below 6 deemed unacceptable) (Goulas and Kontominas 2007).

### 2.12 Texture Properties Evaluation of Fillets

Texture analysis was performed on the anterior region of fillets (between the head and dorsal fin, above the lateral line) using a texture analyzer (Model TA.XT Plus; Stable Micro Systems, UK) equipped with a 10 kN load cell. Cubes (20 × 20 × 20 mm) were cut parallel to the muscle fibers and compressed to 25% of their original height using a cylindrical aluminum probe (diameter: 50.8 mm; Model TAPCY10; Copa Pazhuhash Research Co., Iran) at a constant speed of 1 mm/s (Xie et al. 2023).

### 2.13 Data Analysis

The study employed a completely randomized design with four treatments and three replications. SPSS 22 was used for statistical analysis. ANOVA and Duncan's test (*p* < 0.05) were used to analyze the effects of treatments and storage time. The Kruskal‐Wallis and Mann–Whitney *U* tests were used to analyze sensory evaluation data.

## 3 Results and Discussion

### 3.1 DPPH Radical Scavenging Properties of Hydrolyzed Extracts From Microalgae *C. vulgaris*

**FIGURE 1:**
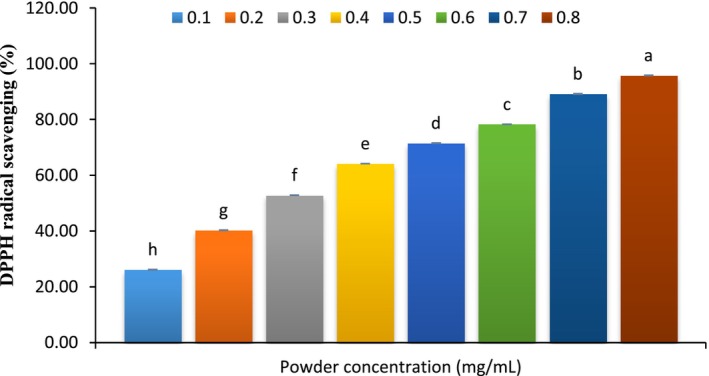
DPPH radical scavenging properties at various concentrations of hydrolyzed extracts from microalga *Chlorella vulgaris* .

DPPH scavenging assays are widely used to evaluate antioxidant activity owing to their speed, cost‐effectiveness, and reliability (Ktari et al. 2013; Onuh et al. 2014). Figure 1 illustrates the antioxidant capabilities of various concentrations of hydrolyzed extracts from the microalga *C. vulgaris* . The findings reveal that the scavenging abilities markedly enhanced as concentrations increased (*p* < 0.05). At a concentration of 0.1 mg/mL of the hydrolyzed extract, the neutralization percentage was 14.26%, the lowest observed value, which progressively rose to a maximum of 79.95% at 0.9 mg/mL. Based on current results, the IC_50_ and IC_80_ values for our samples were determined to be 0.30 mg/mL and 0.61 mg/mL, respectively. These values were utilized, in conjunction with a chitosan solution, to create active coatings intended for the preservation of rainbow trout fillets during refrigeration. As depicted in the results, other studies have also shown a concentration‐dependent increase in the antioxidant properties of hydrolyzed proteins (Mousaie et al. 2022; Yu et al. 2016). For instance, Yu et al. (2016) explored the antioxidant properties of hydrolyzed proteins from the microalga Spirulina and found significant increases at higher concentrations, aligning with our results. However, in their samples, only a minimal increase was reported at a concentration of 5 mg/mL, which differs from our findings. Additionally, it has been noted that low molecular weight peptides in hydrolyzed proteins correlate with a notable enhancement in antioxidant activity, which is influenced by the extent of hydrolysis (Naghdi, Lorenzo, et al. 2023). The functional side chains (R groups) of these lower molecular weight peptides may demonstrate superior antioxidant activity compared to those of higher molecular weight peptides (Naghdi, Rezaei, et al. 2023). Recent studies have highlighted the significant influence of peptide molecular weight on antioxidant performance. Research by Mousaie et al. (2022) demonstrated that smaller peptides possess enhanced antioxidant activity, attributed to their increased ability to interact with and neutralize free radicals. This finding aligns closely with our work, as the composition of the hydrolysates directly contributes to the improved antioxidant efficacy of the chitosan‐based coatings.

### 3.2 Quality Changes in Coated Fish Fillets

#### 3.2.1 Total Volatile Basic Nitrogen (TVBN)

**FIGURE 2:**
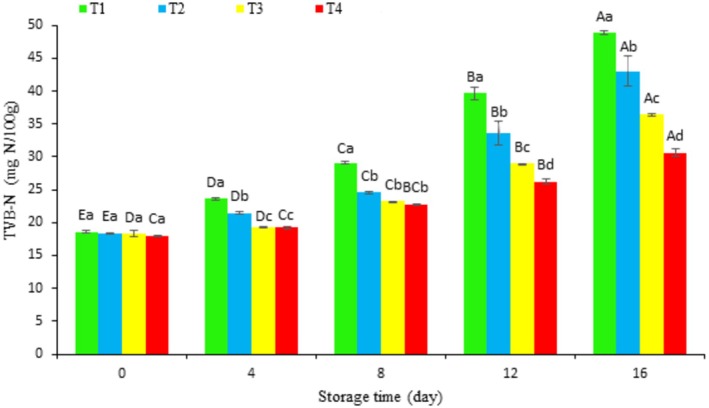
Changes in total volatile basic nitrogen (TVBN) of fish fillets during refrigerated storage. Uppercase letters indicate significant differences over the storage period within each treatment, whereas lowercase letters denote significant differences between the values of different treatments on each specific day.

The total volatile basic nitrogen (TVBN) content was measured in rainbow trout fillets coated with chitosan (T2), chitosan containing IC_50_ hydrolyzed extract (T3), and chitosan containing IC_80_ hydrolyzed extract (T4), and compared to uncoated control (T1) during refrigerated storage and the results were presented in Figure 2. Initially, all samples had a TVBN value of ~18 mg/100 g. Over time, TVBN increased significantly (*p* < 0.05) in all groups, reaching final values of 48.87 mg/100 g, 43.02 mg/100 g, 36.45 mg/100 g, and 30.61 mg/100 g for T1, T2, T3 and T4, respectively. The T3 and T4 coatings showed no significant difference in TVBN increase from Day 0 to 4 (*p* > 0.05). Both T3 and T4 coatings consistently resulted in significantly lower TVBN values than controls throughout storage (Days 4, 8, 12, and 16), with the T4 coating performing better than the T3 coating (*p* < 0.05). Based on a 35 mg/100 g acceptability threshold (Orak and Kayışoğlu 2008), the control group (T1) exceeded this limit by Day 8, whereas the coated groups remained below it. Only T4 group stayed below the limit for the entire storage period. As a key quality indicator, TVBN levels reflect enzymatic and bacterial degradation of proteins, which generate volatile bases and off‐flavors (Hamzeh and Rezaei 2012; Li et al. 2020). Storage conditions and hygiene further modulate this process (Duan et al. 2010). The lower TVBN in coated samples likely reflects the coatings' ability to inhibit bacterial growth (Mehdizadeh et al. 2020; Naghdi, Lorenzo, et al. 2023). These findings align with previous studies showing the effectiveness of various coatings, including gelatin‐based coatings on shrimp (Mirzapour‐Kouhdasht and Moosavi‐Nasab 2020), chitosan films with essential oils (Alparslan and Baygar 2017), and chitosan with hydrolyzed protein on tuna (Kumar et al. 2023), in controlling TVBN and extending shelf life. Also, our findings consistence with Kumar et al. (2023), who demonstrated that protein hydrolysate‐enriched coatings effectively suppress bacterial proliferation and prolong seafood shelf life. This correlation is further supported by Naghdi, Lorenzo, et al. (2023), whose work revealed that natural bioactive compounds in coatings simultaneously reduce microbial contamination and improve sensory characteristics—consistent with our observed dual benefits of *C. vulgaris* hydrolysates in both preservation and quality maintenance. The protease‐inhibiting capacity of our hydrolysates, as evidenced in this study, reveals an additional preservation mechanism. This observation corroborates Pezeshk et al. (2019)'s emphasis on proteolytic enzyme suppression as a critical factor in sustaining fish quality during storage, providing mechanistic validation for our shelf‐life extension results.

#### 3.2.2 pH Changing of Fish Fillets During Cold Storage

**FIGURE 3:**
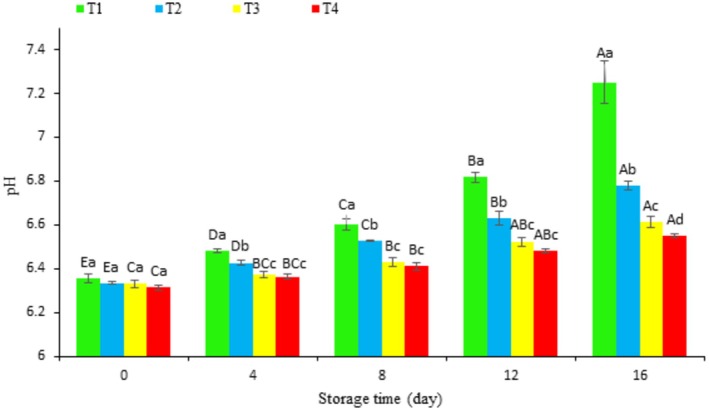
Changes in pH of fish fillets during refrigerated storage. Uppercase letters indicate significant differences over the storage period within each treatment, whereas lowercase letters denote significant differences between the values of different treatments on each specific day.

During refrigeration, the pH of all treated fish fillets increased (*p* < 0.05, Figure 3). This increase was significantly less pronounced in fillets coated with chitosan containing hydrolyzed extract (T3 and T4) compared to the control and chitosan‐only coatings (*p* < 0.05). Starting at a similar pH of approximately 6.3, all samples saw an increase during storage, reaching final values of 7.25 (T1), 6.78 (T2), 6.61 (T3), and 6.55 (T4). Although the T3 and T4 treatments showed no significant pH differences for most of the storage period, a significant difference emerged by Day 16. The pH increase is likely owing to bacterial breakdown of proteins, fats, and carbohydrates, resulting in the production of alkaline substances like ammonia and trimethylamine (Naghdi et al. 2022). The T4 coating was most effective at minimizing pH changes, mirroring its effectiveness in controlling TVBN, suggesting a strong inhibitory effect on bacterial activity. Although the T3 and T4 treatments performed similarly for the first 12 days, all treatments ultimately showed significantly different pH levels by Day 16 (*p* < 0.05).

#### 3.2.3 TBA Change During Refrigerated Storage of Coated Fish Fillets

**FIGURE 4:**
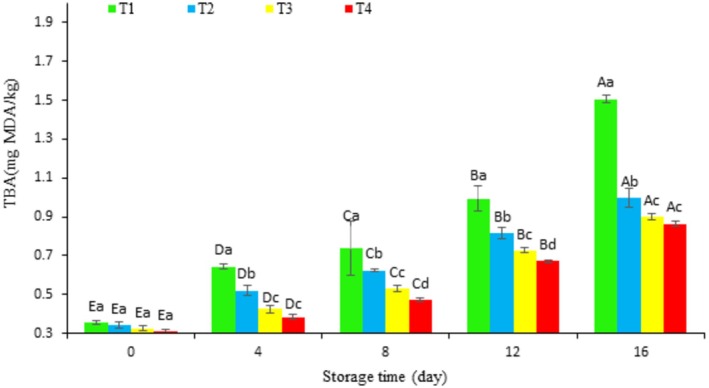
Changes in TBA value of fish fillets during refrigerated storage. Uppercase letters indicate significant differences over the storage period within each treatment, whereas lowercase letters denote significant differences between the values of different treatments on each specific day.

Figure 4 shows a significant increase in the thiobarbituric acid reactive substances (TBARS) index for all treatments throughout the storage period (*p* < 0.05). However, this increase was significantly smaller in the coated samples compared to the control (*p* < 0.05). At the end of storage, the TBARS values were lower in all coated groups, including 0.99 mg MDA/kg (T2), 0.90 mg MDA/kg (T3), and 0.86 mg MDA/kg (T4), compared to 1.5 mg MDA/kg in the T1 group. Although the T3 and T4 treatments showed significant differences in TBARS values for most of the storage period (*p* < 0.05), there was no significant difference between these two treatments on the final day (Day 16, *p* > 0.05). According to the findings, TBA values for all samples demonstrated an upward trend, showing significant variations in the increase on different treatment days (*p* < 0.05). This rise can be linked to the dehydration of the fish, which enhances the oxidation of free fatty acids, along with the presence of free iron and other peroxidants (Alparslan and Baygar 2017; Bonilla et al. 2018; Jasour and Rahimabadi 2011). Malondialdehyde (MDA), a three‐carbon compound, is acknowledged as the main carbonyl generated from the autoxidation of long‐chain polyunsaturated fats. The TBA measurement relies on the interaction between one molecule of MDA and two molecules of TBA, resulting in a pink hue that can be quantified with a spectrophotometer (Naghdi et al. 2022). The formation of these secondary oxidation products contributes to the further degradation of fats and proteins since they can bind with proteins, diminishing their functional properties (Bonilla et al. 2018; Naghdi et al. 2022). A satisfactory TBA level should be below 3 mg of MDA per kilogram for high‐quality products, and it should not surpass 5 mg/kg (Ucak et al. 2020). This limit was consistently exceeded in all treatments during the storage duration. In this research, fish rich in both saturated and unsaturated fatty acids were found to be more prone to lipid oxidation, raising significant quality issues, such as off‐flavors and odors (rancidity) (Mohammadi et al. 2021). Furthermore, alterations in nutritional value, color, and texture could be expected throughout the storage period (Parvathy et al. 2018). Samples that were coated with chitosan and chitosan combined with hydrolyzed protein showed lower TBA values during the storage period, likely owing to the coatings' ability to restrict oxygen exposure (Senadheera et al. 2021). Notable differences were also observed among the coated fillets, with those treated with chitosan (T2) and hydrolyzed protein (T3 and T4) exhibiting the lowest TBA values. These observations can be attributed to the antioxidant properties of the hydrolyzed proteins and chitosan coatings (Muñoz‐Tebar et al. 2023). This is consistent with the findings by Parvathy et al. (2018), who used hydrolyzed proteins from *Euthynnus affinis* as antioxidants for preserving sardines under cold storage. Additionally, Senadheera et al. (2021) found that hydrolysates derived from various parts of sea cucumbers using different enzymes, such as alcalase, flavorzyme, and corolase, demonstrated significant TBA inhibitory activity when compared to butylated hydroxytoluene (BHT). Finally, it is important to consider the relationship between TBA values and sensory acceptability. Although TBA values remained below critical levels, sensory evaluation indicated a gradual decrease in overall acceptability, suggesting that other factors, such as texture, color, and odor also contribute to consumer perception of quality.

#### 3.2.4 Changing in PV of Coated Fish Fillets During Storage

**FIGURE 5:**
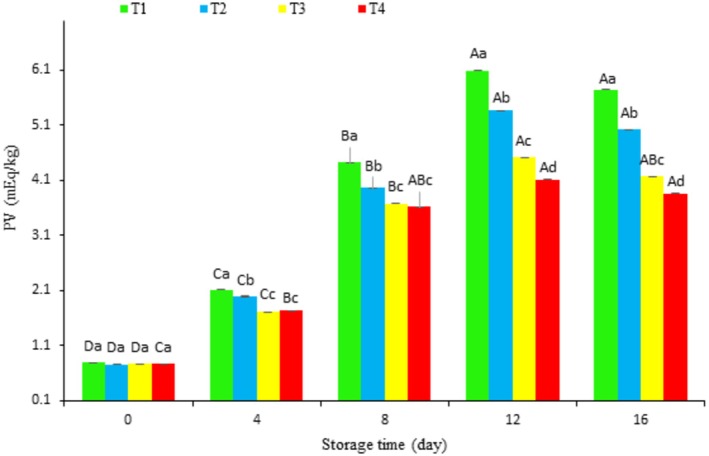
Changes in PV of fish fillets during refrigerated storage. Uppercase letters indicate significant differences over the storage period within each treatment, whereas lowercase letters denote significant differences between the values of different treatments on each specific day.

Changes in peroxide value (PV), a measure of primary lipid oxidation, were measured during refrigerated storage for coated fish fillet samples and the results are shown in Figure 5. Initially (Day 0), all fillet samples had PV values below 0.26 mEq/kg, with no significant differences between treatments (*p* > 0.05). PV increased significantly in all samples up to Day 12, reaching peak values of 2.23, 1.86, 1.46, and 1.00 mEq/kg in treatments of T1, T2, T3, and T4, respectively (*p* < 0.05). After Day 12, PV decreased in all samples. The chitosan containing IC_80_ coating (T4) demonstrated the smallest increase in PV, roughly half that of the control (T1). Except for Day 0, significant differences in PV were observed between treatments throughout the storage period (*p* < 0.05). Increasing PV is a key indicator of declining quality and fat instability. The natural balance between prooxidants and antioxidants in fish is disrupted postmortem, leading to oxidative spoilage (de Jorge Gouvêa et al. 2023; Secci and Parisi 2016). Hydroperoxides, formed as intermediary products, can decompose into secondary oxidation products (de Jorge Gouvêa et al. 2023) or react with other components in the fish, causing undesirable odors and color changes (Hematyar et al. 2019). Although the acceptable limit for PV in seafood is 5 mEq/kg (Piedrahíta Márquez et al. 2019), all samples in this study remained below this threshold. The initial PV increase (up to Day 12) is attributed to hydroperoxide formation, whereas the subsequent decrease is likely owing to hydroperoxide degradation (Naghdi et al. 2022). The coatings, especially those incorporating hydrolyzed protein, effectively reduced PV. The T4 coating performed best, followed by T3 and chitosan alone (T2), with the control (T1) showing the highest PV. This suggests that the coatings limit oxygen exposure, thus reducing oxidation (Weng et al. 2009). These results are supported by other studies showing the protective effects of chitosan coatings containing hydrolyzed protein against lipid oxidation in fish fillets (Kumar et al. 2023). The antioxidant properties of amino acids like cysteine, tyrosine, tryptophan, and histidine within the coating materials likely contribute to free radical scavenging (Kang et al. 2013). Furthermore, the antioxidant activity of hydrolyzed proteins, chitosan, and starch has been well‐documented (Alparslan and Baygar 2017; Naghdi, Lorenzo, et al. 2023; Yıldırım‐Yalçın et al. 2021). The latter study showed reduced peroxide levels in rainbow trout coated with peptides from bighead carp, highlighting the potential of these peptides as antioxidants. The observed decline in peroxide values after Day 12 likely reflects hydroperoxide decomposition into secondary oxidation products, consistent with Hematyar et al.'s (2019) findings. This pattern suggests that while early‐stage oxidation occurs, the coated samples demonstrate enhanced lipid stabilization capacity over prolonged storage. Such stabilization is crucial for maintaining both sensory attributes and nutritional quality throughout the product's shelf life.

**FIGURE 6:**
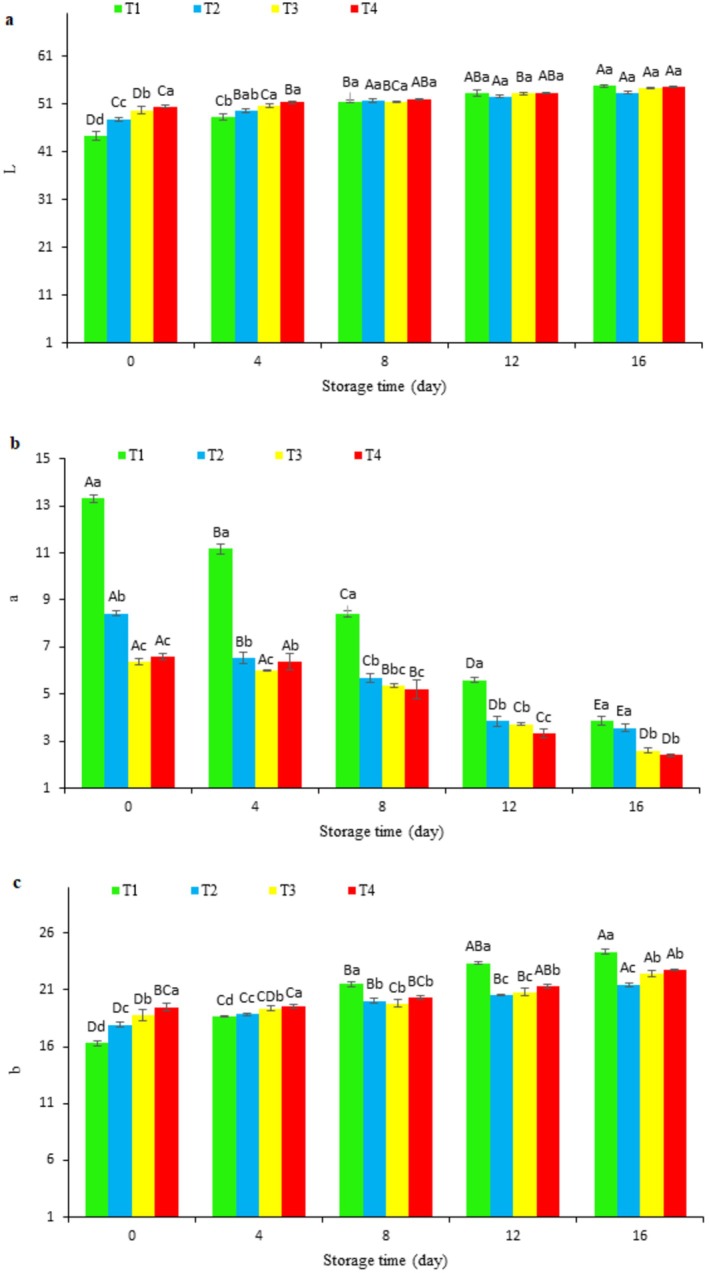
Changes in lab indexes of fish fillets during refrigerated storage. Uppercase letters indicate significant differences over the storage period within each treatment, whereas lowercase letters denote significant differences between the values of different treatments on each specific day.

#### 3.2.5 Color Change of Coated Fish Fillets During Storage

The evaluation of color variations in both coated and uncoated fish fillets during refrigerated storage utilized CIE L* (lightness), a* (redness), and b* (yellowness) metrics. These variations were examined in relation to oxidative processes and sensory quality, building on prior research in this area (Figure 6). The control group (T1) exhibited maximum values of L*, a*, and b* at 54.74, 13.31, and 24.34, respectively, consistent with oxidative discoloration trends documented by Papuc et al. (2017) in their overview of meat oxidation mechanisms. The increase in a* values (redness) observed in control samples corresponds with the findings of Lynch and Faustman (2000), who indicated that lipid oxidation products can facilitate myoglobin oxidation, resulting in undesirable color alterations in meat products. Our study extends these insights to fish fillets, demonstrating that chitosan coatings enhanced with *C. vulgaris* hydrolysate effectively counteract this issue, akin to the protective benefits noted by Arancibia et al. (2015) in chitosan‐coated shrimp. Lightness (L*) exhibited a slight increase over time, with the T4 coating preserving the highest values during the initial storage period. This aligns with the results of Farajzadeh et al. (2016), who observed enhanced lightness in chitosan‐coated shrimp, whereas our research further indicates that the inclusion of algal hydrolysates amplifies this effect. The sustained L* values in coated samples were strongly linked to elevated sensory scores for appearance, corroborating the sensory‐color associations identified by Ojagh et al. (2010) in trout coated with chitosan and cinnamon oil. The increase in yellowness (b*) was most significant in control samples, mirroring the oxidative degradation trends outlined by Secci and Parisi (2016) in their review of lipid oxidation in fish products. Notably, our hydrolysate‐enriched coatings decreased b* values by 30% compared to control samples, surpassing the effects noted by Alparslan and Baygar (2017) for chitosan films infused with orange peel oil. This enhancement is likely attributable to the robust antioxidant properties of *C. vulgaris* peptides, which we previously characterized through DPPH assays. The correlation between instrumental color measurements and sensory evaluations highlighted critical consumer perception thresholds. Control samples surpassed the critical a* (> 10) and b* (> 20) values linked to decreased purchase intent in seafood (de Oliveira Filho et al. 2019) by Day 8, coinciding with sensory scores falling below acceptable levels. In contrast, coated samples maintained acceptable coloration throughout the storage period, similar to the preservation effects achieved by Shah Hosseini et al. (2021) using pullulan films enriched with plant extracts. These findings expand upon our earlier results regarding the antioxidant capacity of *C. vulgaris* hydrolysates and their implications for lipid oxidation. The stabilization of color in T4 samples aligns with its superior performance in reducing TBARS and PV values, illustrating how oxidative processes impact multiple quality parameters concurrently. This multifaceted protection reflects the extensive preservation effects reported by Naghdi, Lorenzo, et al. (2023) in their study of fish protein hydrolysate coatings. Although the coatings effectively delayed color degradation, the initial slight reduction in L* values upon application requires further scrutiny. This phenomenon was less pronounced than the effects documented by Mehdizadeh et al. (2020) in chitosan‐starch films, suggesting our formulation achieves improved optical characteristics. Future research could focus on optimizing coating transparency while retaining antioxidant efficacy, potentially building on methodologies developed by Muñoz‐Tebar et al. (2023) for bioactive chitosan composites. The strong correlation between our instrumental color data and sensory evaluations validates the use of CIE L*a*b* measurements as reliable indicators of quality, as proposed by Cheng et al. (2014) in their review of fish freshness assessment methods. Our results indicate that chitosan coatings containing *C. vulgaris* hydrolysate can effectively preserve the visual appeal of trout fillets while addressing multiple spoilage pathways, offering advantages over single‐function approaches like those described by Safari et al. (2018) for antioxidant‐treated bighead carp.

#### 3.2.6 Changes in Bacterial Levels of Fish Fillets During Refrigerated Storage

Microbial changes in fish fillets during refrigerated storage were assessed by measuring mesophilic and psychrotrophic bacterial counts (Table 1). Initial mesophilic bacterial loads were below 3.5 × 10^4^ CFU/g in both control and coated samples, indicating acceptable initial conditions. However, these counts increased significantly over time in all treatments (*p* < 0.05). The highest counts, 2.6 × 10^7^ CFU/g (mesophilic) and 4.2 × 10^7^ CFU/g (psychrotrophic), were observed in the uncoated control, significantly higher than the coated samples containing hydrolyzed extract (*p* < 0.05). The T4 coating showed the lowest initial microbial counts and the smallest increase over time for both mesophilic and psychrotrophic bacteria (*p* < 0.05). Interestingly, whereas psychrotrophic counts initially increased in all samples, the T3 and T4 coatings showed a decrease in psychrotrophic bacteria after Day 8 (*p* < 0.05). The IC_80_ coating (T4) suppressed psychrotrophic bacterial growth by 50% compared to the control (*p* < 0.05), confirming hydrolysate antimicrobial efficacy. These results are consistent with previous studies demonstrating the antimicrobial effects of coatings containing hydrolyzed fish proteins. The observed increases in TVBN (Figures 2 and 4), and PV (Figure 5) were closely correlated with microbial proliferation (Table 1), particularly in uncoated fillets (T1). For instanc, the sharp rise in TVBN (48.87 mg/100 g in T1 vs. 30.61 mg/100 g in T4 by Day 16) paralleled the 2‐log higher bacterial counts in T1, suggesting microbial deamination of proteins as the primary driver (Li et al. 2020). However, the slower TVBN increase in coated groups (T3–T4) implies that *C. vulgaris* hydrolysate also suppressed endogenous proteases (e.g., cathepsins), as evidenced by lower TVBN even when bacterial loads were similar across treatments on Day 8 (*p* > 0.05). Although lipid oxidation (TBA/PV) is typically enzymatically initiated (e.g., via lipoxygenase), the coatings' antioxidant properties (IC_50_/IC_80_) likely directly quenched free radicals, decoupling oxidation from microbial activity. This is supported by the T4 group's lower TBA (0.86 vs. 1.5 mg MDA/kg in T1) despite comparable psychrotrophic counts on Day 12 (*p* > 0.05). Naghdi, Lorenzo, et al. (2023) reported antimicrobial activity in rainbow trout fillets coated with starch and starch‐alginate containing hydrolyzed fish protein. Similarly, de Oliveira Filho et al. (2019) found antimicrobial activity against both Gram‐positive and Gram‐negative bacteria in active packaging films based on alginate and hydrolyzed cottonseed protein. Mirzapour‐Kouhdasht and Moosavi‐Nasab (2020) also showed that gelatin hydrolysate coatings inhibited microbial growth in shrimp. Furthermore, Hu et al. (2013) observed similar antimicrobial effects in common carp fillets coated with hydrolyzed silver carp protein and fish skin gelatin. The antibacterial activity of hydrolyzed proteins depends on several factors, including amino acid composition, sequence, molecular weight, and structural characteristics, such as helicity, hydrophobicity, hydrophobic moment, and charge, as well as the specific bacteria targeted (Pezeshk et al. 2019; Qara and Habibi 2018; Shaibani et al. 2020; Wald et al. 2016). The interaction between cationic peptides and negatively charged components of bacterial cell walls (e.g., lipoteichoic acid in Gram‐positive bacteria, phosphate groups in Gram‐negative bacteria) contributes to their antimicrobial action (Pezeshk et al. 2019). Peptides with high hydrophobic amino acid content (up to 50%) can exhibit strong antibacterial properties, as increased hydrophobicity enhances membrane permeability, disrupting bacterial cell membranes (Shaibani et al. 2020; Wald et al. 2016). The combination of chitosan and hydrolysate enhanced electrostatic interactions with bacterial surfaces, as evidenced by T4's 50% greater suppression of psychrotrophs than T2 (chitosan‐only). This aligns with studies showing that chitosan‐peptide composites exhibit broader‐spectrum activity than either component alone (da Rocha et al. 2018).

**TABLE 1:** Changes in mesophilic and cryophilic bacterial load of coated and uncoated fillets during refrigerated storage (CFU/g).

Day	Bacetria	T1	T2	T3	T4
0	Mesophile	3.5 × 104 ± 0.5 Ea	3 × 104 ± 1.0 Eb	3 × 104 ± 0.0 Eb	2 × 104 ± 1.0 Ec
	Cryophilic	2.5 × 104 ± 1 Ec	3.5 × 104 ± 1 Ea	3 × 104 ± 5.5 Eb	1 × 104 ± 5.5 Ed
4	Mesophile	17 × 104 ± 5.0 Da	14.5 × 104 ± 0.5 Db	9 × 104 ± 2.0 Dc	8 × 104 ± 1.0 Dc
	Cryophilic	30.5 × 104 ± 2.5 Da	22 × 104 ± 2.0 Db	12.5 × 104 ± 1.5 Dc	12 × 104 ± 1.0 Dc
8	Mesophile	18.5 × 105 ± 2.5 Ca	9 × 105 ± 0.0 Cb	5 × 105 ± 1.0 Cc	3.5 × 105 ± 1.5 Cc
	Cryophilic	29 × 105 ± 2.5 Ca	13 × 105 ± 0.0 Cb	10.5 × 105 ± 1.0 Cb	8 × 105 ± 1.5 Cc
12	Mesophile	34 × 106 ± 2.0 Ba	7.5 × 106 ± 3.0 Bb	3.5 × 106 ± 0.5 Bb	4.5 × 106 ± 1.0 Bb
	Cryophilic	39.5 × 106 ± 2.5 Ba	13.5 × 106 ± 2.5 Bb	7.5 × 106 ± 0.5 Bc	3 × 106 ± 1.0 Bd
16	Mesophile	26 × 107 ± 2.0 Aa	17 × 107 ± 2.0 Ab	8 × 107 ± 1.0 Ac	4 × 107 ± 0.0 Ad
	Cryophilic	42 × 107 ± 4.0 Aa	22.5 × 107 ± 0.5 Ab	11.5 × 107 ± 1.5 Ac	5 × 107 ± 2.0 Ad

*Note:* Uppercase letters indicate significant differences over the storage period within each treatment, whereas lowercase letters denote significant differences between the values of different treatments on each specific day.

### 3.3 Texture Properties Analysis of Coated Fish Fillets During Refrigerated Storage

Changes in texture, specifically hardness and adhesiveness, were analyzed in coated and uncoated fish fillets during refrigerated storage and the results are presented in Table 2. Both indices changed significantly over time (*p* < 0.05), with some samples exhibiting consistent trends and others showing irregular patterns. Hardness generally increased from Day 0 to Day 4 in all but the chitosan‐coated sample. The control and both chitosan containing hydrolyzed extract (T3 and T4) samples showed increases, with the T4 group exhibiting a statistically significant increase. The highest hardness value (558.85 N) was observed in the T4 group on Day 4, significantly different from all other samples and time points (*p* < 0.05). Adhesiveness also changed significantly, reaching a maximum value of 14.73 g/mm in the T4 group on Day 16 (*p* < 0.05). However, the overall trend for adhesiveness was irregular, both between treatments and within each treatment over time. These findings contrast with some previous studies reporting a decrease in hardness during storage, with the lowest values observed at the end of the storage period (Cai et al. 2015, 2014). In this study, the lowest hardness was found in the control group, whereas the chitosan containing IC_80_ coating (T4) exhibited the highest hardness (*p* < 0.05). Variations in hardness can be attributed to several factors, including coating type, fish age and size, nutritional composition, and batch‐to‐batch variability (Cheng et al. 2014; Feng et al. 2017). However, the irregular trends observed in adhesiveness suggest that the interactions between the coating and the fish fillet are complex. Although T4 showed a maximum adhesiveness value of 14.73 g/mm, this irregularity may reflect variations in moisture content and protein integrity over time, as noted by Mahto et al. (2015). The combination of chitosan and hydrolysate not only acts as a physical barrier but also may influence the water‐holding capacity of the fillets, affecting texture attributes. The highest adhesiveness value was observed in the T4 group (14.73 ± 0.05 g/mm, *p* < 0.05). Although this study observed an irregular trend in adhesiveness, other studies have shown a general decrease in adhesiveness during storage (*p* < 0.05). Changes in hardness and adhesiveness may be related to the degradation of myofibrillar proteins, which comprise 70%–80% of total fish muscle protein (Feng et al. 2017). Decreased hardness and increased adhesiveness can indicate quality deterioration and reduced consumer acceptability (Mahto et al. 2015). Díaz‐Tenorio et al. (2007) suggested that differences in hardness may be related to variations in protein structure between samples. The lower hardness values observed in the uncoated control group may be owing to proteolytic activity from endogenous or microbial proteases and collagenases.

**TABLE 2:** Changes in the textural indicators of hardness and stickiness of the coated fillets stored at refrigerator temperature.

Storage days		T1	T2	T3	T4
0	Hardness (N)	435.65 ± 36.15 Ac	516.00 ± 29.00 Aa	420.95 ± 3.15 Ad	497.15 ± 32.85 Bb
	Adhesiveness (g/mm)	4.25 ± 1.98 Dc	11.58 ± 3.20 Ab	14.14 ± 2.30 Aa	14.73 ± 0.05 Aa
4	Hardness (N)	436.70 ± 44.30 Ab	360.10 ± 47.20 Bc	427.70 ± 107.9 Ab	558.85 ± 3.75 Aa
	Adhesiveness (g/mm)	6.93 ± 0.89 Cc	5.47 ± 0.34 Dd	8.00 ± 0.96 Ba	7.19 ± 0.85 Bb
8	Hardness (N)	307.30 ± 23.30 Bc	305.75 ± 52.85 Cbc	311.55 ± 35.35 Bb	344.90 ± 56.10 Ca
	Adhesiveness (g/mm)	9.67 ± 3.94 Aa	6.37 ± 2.69 Cd	7.94 ± 1.32 Bc	8.94 ± 3.03 Bb
12	Hardness (N)	236.20 ± 21.50 Cd	360.90 ± 32.60 Bb	323.75 ± 10.95 Bc	373.25 ± 29.65 Ca
	Adhesiveness (g/mm)	8.63 ± 0.15 Ba	7.70 ± 1.65 Bc	8.25 ± 3.04 Bb	7.79 ± 3.53 Bc
16	Hardness (N)	209.75 ± 26.65 Dc	270.25 ± 9.85 Db	276.65 ± 4.15 Cb	308.55 ± 14.85 Da
	Adhesiveness (g/mm)	9.48 ± 1.24 Aa	4.54 ± 1.66 Eb	4.54 ± 0.86 Cb	4.44 ± 2.06 Cb

*Note:* The capital letters indicate significant differences over the storage period within each treatment, whereas small letters denote significant differences between the values of different treatments on each specific day.

### 3.4 Sensory Evaluation of Coated Samples

Sensory evaluation of coated and uncoated fish fillets was conducted during refrigerated storage, assessing taste, texture, aroma, color, and overall acceptance (Table 3). Significant changes in all sensory attributes were observed over time (*p* < 0.05). Initially (Day 0), the control group received the highest scores for taste, texture, color, and overall acceptance, whereas the chitosan‐coated group (T3 and T4) scored highest for aroma (*p* < 0.05). Sensory quality declined significantly over time for all samples, but the decline was slower in the fillets coated with chitosan containing hydrolyzed extract. For instance, the taste score for the control group dropped from 9.67 on Day 0 to 4.53 on Day 8, whereas the T4 group showed a smaller decrease, from 9.40 to 7.07. After Day 0, the samples consistently ranked (from highest to lowest sensory scores) as follows: T4, T3, T2, and T1. By Day 16, there was no significant difference in overall acceptance between the T3 and T4 groups, but both were significantly higher than the T2 and T1 groups (*p* < 0.05). Interestingly, the initial overall acceptance scores for all coated groups (T2: 9.80 ± 0.40; T2: 9.20 ± 0.65; T3: 9.07 ± 0.68) were lower than the T1 group (9.80 ± 0.40), suggesting that the coatings may have initially negatively impacted sensory perception. Throughout storage, the chitosan containing hydrolyzed protein coatings consistently received the highest sensory scores, indicating their effectiveness in preserving sensory quality. This aligns with the observed improvements in microbial and chemical stability discussed in other sections. These results are consistent with Mirzapour‐Kouhdasht and Moosavi‐Nasab (2020), who reported that coated fillets maintained higher sensory scores. Although initial sensory scores were slightly reduced in coated samples, the T4 treatment showed progressive improvement during storage, mirroring Alotaibi and Tahergorabi's (2018) observations regarding natural antioxidant coatings. Notably, our coatings successfully maintained critical sensory attributes like color and texture—key determinants of consumer acceptance—supporting de Oliveira Filho et al.'s (2019) emphasis on visual appeal as a crucial quality parameter in seafood marketing. However, unlike our study, their gelatin‐based coatings did not negatively impact initial sensory quality. Other studies have also demonstrated the sensory benefits of incorporating hydrolyzed proteins and other active ingredients into coatings. Parvathy et al. (2018) found that hydrolyzed protein from fish waste improved the antioxidant capacity and preservation of sardines during refrigerated storage. Alotaibi and Tahergorabi (2018) showed that thyme oil in starch coatings enhanced the sensory attributes of shrimp. Similarly, Kumar et al. (2023) reported that coatings containing hydrolyzed fish protein, chitosan, and clove essential oil effectively preserved the sensory quality of tuna fillets.

**TABLE 3:** Changes in the textural indicators of hardness and stickiness of the coated fillets storage at refrigerator temperature.

Storage day	Treatments	Color	Aroma	Texture	Taste	Overall acceptance
0	T1	9.93 ± 0.25 Aa	9.47 ± 0.72 Ab	9.73 ± 0.44 Aa	9.67 ± 0.60 Aa	9.80 ± 0.40 Aa
	T2	9.00 ± 0.73 Ab	9.60 ± 0.61 Aa	9.53 ± 0.50 Ac	9.53 ± 0.72 Ab	9.53 ± 0.50 Ab
	T3	8.73 ± 0.85 Ac	9.33 ± 0.79 Ac	9.53 ± 0.62 Ac	9.40 ± 0.71 Ac	9.20 ± 0.65 Ac
	T4	8.53 ± 0.96 Ad	9.20 ± 0.83 Ad	9.60 ± 0.61 Ab	9.40 ± 0.61 Ac	9.07 ± 0.68 Ad
4	T1	7.27 ± 0.68 Ba	6.27 ± 0.77 Bb	7.27 ± 0.93 Bb	6.73 ± 0.85 Bc	6.67 ± 0.70 Bc
	T2	7.20 ± 0.65 Bb	6.93 ± 0.77 Bb	7.13 ± 0.88 Bc	7.53 ± 0.88 Bb	7.27 ± 0.57 Bab
	T3	7.33 ± 0.60 Ba	7.47 ± 0.81 Ba	7.40 ± 0.61 Bb	8.33 ± 0.70 Ba	7.60 ± 0.49 Ba
	T4	7.20 ± 0.75 Bb	8.07 ± 0.57 Ba	8.07 ± 0.68 Ba	8.47 ± 0.72 Ba	7.93 ± 0.44 Ba
8	T1	4.67 ± 0.94 Cc	3.53 ± 0.81 Cd	4.67 ± 0.70 Cc	4.53 ± 1.02 Cc	4.07 ± 0.68 Bd
	T2	5.07 ± 0.77 Cab	4.07 ± 0.77 Cc	5.67 ± 0.70 Cb	5.80 ± 0.83 Cc	5.20 ± 0.54 Bc
	T3	5.07 ± 0.68 Cab	4.73 ± 1.00 Cb	6.33 ± 0.70 Ca	6.67 ± 0.47 Cab	5.73 ± 0.44 Bb
	T4	5.27 ± 0.57 Ca	5.47 ± 0.96 Ca	6.80 ± 0.54 Ca	7.07 ± 0.68 Ca	6.27 ± 0.57 Ba
12	T1	1.67 ± 0.60 Dc	1.27 ± 0.44 Dd	1.93 ± 0.57 Da	1.73 ± 0.68 Dd	1.73 ± 0.57 Cc
	T2	2.13 ± 0.72 Db	1.47 ± 0.50 Dc	2.33 ± 0.87 Db	3.07 ± 0.68 Db	2.47 ± 0.50 Cb
	T3	2.13 ± 0.62 Db	1.87 ± 0.81 Db	2.73 ± 0.93 Db	3.53 ± 0.72 Da	2.87 ± 0.62 Cb
	T4	2.40 ± 0.49 Da	2.13 ± 0.72 Da	3.13 ± 0.96 Da	3.60 ± 1.02 Da	3.00 ± 0.52 Ca
16	T1	1.27 ± 0.44 Dc	1.00 ± 0.00 Dd	1.20 ± 0.40 Dc	1.00 ± 0.00 Eb	1.13 ± 0.34 Cbc
	T2	1.27 ± 0.44 Dc	1.13 ± 0.34 Dc	1.33 ± 0.47 Db	1.13 ± 0.34 Eb	1.27 ± 0.44 Db
	T3	1.33 ± 0.47 Dab	1.27 ± 0.44 Db	1.60 ± 0.49 Da	1.33 ± 0.47 Ea	1.53 ± 0.50 Da
	T4	1.40 ± 0.49 Da	1.33 ± 0.47 Da	1.67 ± 0.49 Da	1.40 ± 0.49 Ea	1.67 ± 0.47 Da

*Note:* The capital letters indicate significant differences over the storage period within each treatment, whereas small letters denote significant differences between the values of different treatments on each specific day.

## 4 Conclusion

In conclusion, this study demonstrates that chitosan coatings enhanced with *C. vulgaris* hydrolysates significantly prolong the shelf life of rainbow trout fillets during refrigerated storage inhibiting both microbial growth and oxidative deterioration. Notably, the IC_80_‐enriched coating (T4) exhibited exceptional performance, maintaining sensory acceptability for up to 16 days while decreasing TBARS values by 43% and TVBN accumulation by 37% compared to uncoated controls. These results underscore the potential of microalgal hydrolysates as natural preservatives in active food packaging systems. Future research should focus on three key areas: (1) Scaling studies to assess industrial feasibility, including the optimization of coating application techniques (e.g., spraying vs. dipping) for high‐throughput processing; (2) conducting long‐term stability trials (beyond 16 days) to ascertain the maximum shelf life extension achievable under commercial refrigeration conditions; and (3) real‐world validation through supermarket display tests to evaluate color stability and consumer acceptance amidst fluctuating temperature conditions. Additionally, further investigations should explore cost–benefit analyses of hydrolysate production and potential synergistic effects with other natural preservatives to enhance commercial viability. These efforts will help bridge the gap between laboratory successes and practical applications in the food industry. This research provides a foundation for the development of sustainable, algae‐based preservation technologies that could significantly reduce food waste while addressing the increasing consumer demand for clean‐label seafood products.

## Author Contributions

**Nadia Yazdani:** formal analysis (equal), writing – original draft (equal). **Sakineh Yeganeh:** supervision (lead). **Mina Esmaeili Kharyeki:** conceptualization (equal), methodology (equal). **Shahab Naghdi:** data curation (equal), writing – review and editing (equal).

## Conflicts of Interest

The authors declare no conflicts of interest.

## Data Availability

The data that support the findings of this study are available from the corresponding author upon reasonable request.

## References

[fsn370378-bib-0001] Abd El‐Hack, M. E. , M. T. El‐Saadony , M. E. Shafi , et al. 2020. “Antimicrobial and Antioxidant Properties of Chitosan and Its Derivatives and Their Applications: A Review.” International Journal of Biological Macromolecules 164: 2726–2744. 32841671 10.1016/j.ijbiomac.2020.08.153

[fsn370378-bib-0002] Abdel‐Naeem, H. H. S. , K. I. Sallam , and N. M. L. Malak . 2021. “Improvement of the Microbial Quality, Antioxidant Activity, Phenolic and Flavonoid Contents, and Shelf Life of Smoked Herring ( *Clupea harengus* ) During Frozen Storage by Using Chitosan Edible Coating.” Food Control 130: 108317.

[fsn370378-bib-0003] Al‐Hammadi, M. , and M. Güngörmüşler . 2024. “New Insights Into *Chlorella vulgaris* Applications.” Biotechnology and Bioengineering 121: 1486–1502. 38343183 10.1002/bit.28666

[fsn370378-bib-0004] Alotaibi, S. , and R. Tahergorabi . 2018. “Development of a Sweet Potato Starch‐Based Coating and Its Effect on Quality Attributes of Shrimp During Refrigerated Storage.” LWT 88: 203–209. 10.1016/j.lwt.2017.10.022.

[fsn370378-bib-0005] Alparslan, Y. , and T. Baygar . 2017. “Effect of Chitosan Film Coating Combined With Orange Peel Essential Oil on the Shelf Life of Deepwater Pink Shrimp.” Food and Bioprocess Technology 10: 842–853. 10.1007/s11947-017-1862-y.

[fsn370378-bib-0006] Arancibia, M. Y. , M. E. López‐Caballero , M. C. Gómez‐Guillén , and P. Montero . 2015. “Chitosan Coatings Enriched With Active Shrimp Waste for Shrimp Preservation.” Food Control 54: 259–266. 10.1016/j.foodcont.2015.02.004.

[fsn370378-bib-0007] Bagheri, R. , R. Izadi Amoli , N. Tabari Shahndasht , and S. R. Shahosseini . 2016. “Comparing the Effect of Encapsulated and Unencapsulated Fennel Extracts on the Shelf Life of Minced Common Kilka (*Clupeonella cultriventris* Caspia) and *Pseudomonas aeruginosa* Inoculated in the Mince.” Food Science & Nutrition 4: 216–222. 27004111 10.1002/fsn3.275 PMC4779485

[fsn370378-bib-0008] Bonilla, F. , A. Chouljenko , V. Reyes , P. J. Bechtel , J. M. King , and S. Sathivel . 2018. “Impact of Chitosan Application Technique on Refrigerated Catfish Fillet Quality.” LWT 90: 277–282. 10.1016/j.lwt.2017.12.010.

[fsn370378-bib-0009] Cai, L. , A. Cao , F. Bai , and J. Li . 2015. “Effect of ε‐Polylysine in Combination With Alginate Coating Treatment on Physicochemical and Microbial Characteristics of Japanese Sea Bass (*Lateolabrax japonicas*) During Refrigerated Storage.” LWT ‐ Food Science and Technology 62: 1053–1059. 10.1016/j.lwt.2015.02.002.

[fsn370378-bib-0010] Cai, L. , X. Li , X. Wu , Y. Lv , X. Liu , and J. Li . 2014. “Effect of Chitosan Coating Enriched With Ergothioneine on Quality Changes of Japanese Sea Bass (*Lateolabrax japonicas*).” Food and Bioprocess Technology 7: 2281–2290. 10.1007/s11947-013-1215-4.

[fsn370378-bib-0011] Cermeño, M. , J. Stack , P. R. Tobin , et al. 2019. “Peptide Identification From a Porphyra Dioica Protein Hydrolysate With Antioxidant, Angiotensin Converting Enzyme and Dipeptidyl Peptidase IV Inhibitory Activities.” Food & Function 10: 3421–3429. 10.1039/C9FO00680J. 31134998

[fsn370378-bib-0012] Chamanara, V. , B. Shabanpour , S. Gorgin , and M. Khomeiri . 2012. “An Investigation on Characteristics of Rainbow Trout Coated Using Chitosan Assisted With Thyme Essential Oil.” International Journal of Biological Macromolecules 50: 540–544. 22305883 10.1016/j.ijbiomac.2012.01.016

[fsn370378-bib-0013] Cheng, J. , D. Sun , Z. Han , and X. Zeng . 2014. “Texture and Structure Measurements and Analyses for Evaluation of Fish and Fillet Freshness Quality: A Review.” Comprehensive Reviews in Food Science and Food Safety 13: 52–61. 33412693 10.1111/1541-4337.12043

[fsn370378-bib-0014] da Rocha, M. , A. Alemán , V. P. Romani , et al. 2018. “Effects of Agar Films Incorporated With Fish Protein Hydrolysate or Clove Essential Oil on Flounder ( *Paralichthys orbignyanus* ) Fillets Shelf‐Life.” Food Hydrocolloids 81: 351–363. 10.1016/j.foodhyd.2018.03.017.

[fsn370378-bib-0015] de Jorge Gouvêa, F. , V. S. de Oliveira , B. J. Mariano , et al. 2023. “Natural Antioxidants as Strategy to Minimize the Presence of Lipid Oxidation Products in Canned Fish: Research Progress, Current Trends and Future Perspectives.” Food Research International 173: 113314. 37803625 10.1016/j.foodres.2023.113314

[fsn370378-bib-0016] de Oliveira Filho, J. G. , J. M. Rodrigues , A. C. F. Valadares , et al. 2019. “Active Food Packaging: Alginate Films With Cottonseed Protein Hydrolysates.” Food Hydrocolloids 92: 267–275. 10.1016/j.foodhyd.2019.01.052.

[fsn370378-bib-0018] Díaz‐Tenorio, L. M. , F. L. García‐Carreño , and R. Pacheco‐Aguilar . 2007. “Comparison of Freezing and Thawing Treatments on Muscle Properties of Whiteleg Shrimp (*Litopenaeus vannamei*).” Journal of Food Biochemistry 31: 563–576. 10.1111/j.1745-4514.2007.00130.x.

[fsn370378-bib-0017] Diaz, C. J. , K. J. Douglas , K. Kang , et al. 2023. “Developing Algae as a Sustainable Food Source.” Frontiers in Nutrition 9: 1029841. 36742010 10.3389/fnut.2022.1029841 PMC9892066

[fsn370378-bib-0019] Dini, I. 2023. “The Potential of Algae in the Nutricosmetic Sector.” Molecules 28: 4032. 37241773 10.3390/molecules28104032 PMC10220586

[fsn370378-bib-0020] Duan, J. , Y. Jiang , G. Cherian , and Y. Zhao . 2010. “Effect of Combined Chitosan‐Krill Oil Coating and Modified Atmosphere Packaging on the Storability of Cold‐Stored Lingcod ( *Ophiodon elongates* ) Fillets.” Food Chemistry 122: 1035–1042. 10.1016/j.foodchem.2010.03.065.

[fsn370378-bib-0080] FAO . 2022. The State of World Fisheries and Aquaculture, 2022 : Towards Blue Transformation, 226–236. FAO.

[fsn370378-bib-0021] Farajzadeh, F. , A. Motamedzadegan , S. A. Shahidi , and S. Hamzeh . 2016. “The Effect of Chitosan‐Gelatin Coating on the Quality of Shrimp ( *Litopenaeus vannamei* ) Under Refrigerated Condition.” Food Control 67: 163–170. 10.1016/j.foodcont.2016.02.040.

[fsn370378-bib-0022] Farsani, O. A. , M. Kordjazi , B. Shabanpour , S. M. Ojagh , and A. Jamshidi . 2018. “The Effect of Antioxidant Properties of Brown Algae (*Iyengaria stellata*) Extract on the Shelf‐Life and Sensory Properties of Rainbow Trout (*Oncorhynchus mykiss*) Fillet Nugget During Frozen Storage (−18°C).” Research and Innovation in Food Science and Technology 7: 149–166. 10.22101/JRIFST.2018.07.17.723.

[fsn370378-bib-0023] Feng, X. , V. K. Ng , M. Mikš‐Krajnik , and H. Yang . 2017. “Effects of Fish Gelatin and Tea Polyphenol Coating on the Spoilage and Degradation of Myofibril in Fish Fillet During Cold Storage.” Food and Bioprocess Technology 10: 89–102. 10.1007/s11947-016-1798-7.

[fsn370378-bib-0024] Goulas, A. E. , and M. G. Kontominas . 2007. “Combined Effect of Light Salting, Modified Atmosphere Packaging and Oregano Essential Oil on the Shelf‐Life of Sea Bream ( *Sparus aurata* ): Biochemical and Sensory Attributes.” Food Chemistry 100: 287–296.

[fsn370378-bib-0025] Hamzeh, A. , and M. Rezaei . 2012. “The Effects of Sodium Alginate on Quality of Rainbow Trout (*Oncorhynchus mykiss*) Fillets Stored at 4 ± 2°C.” Journal of Aquatic Food Product Technology 21, no. 1: 14–21. 10.1080/10498850.2011.579384.

[fsn370378-bib-0026] Harnedy, P. A. , and R. J. FitzGerald . 2013. “In Vitro Assessment of the Cardioprotective, Anti‐Diabetic and Antioxidant Potential of *Palmaria palmata* Protein Hydrolysates.” Journal of Applied Phycology 25: 1793–1803.

[fsn370378-bib-0027] Hathwar, S. C. , B. Bijinu , A. K. Rai , and B. Narayan . 2011. “Simultaneous Recovery of Lipids and Proteins by Enzymatic Hydrolysis of Fish Industry Waste Using Different Commercial Proteases.” Applied Biochemistry and Biotechnology 164: 115–124. 10.1007/s12010-010-9119-5. 21057982

[fsn370378-bib-0028] Hematyar, N. , T. Rustad , S. Sampels , and T. Kastrup Dalsgaard . 2019. “Relationship Between Lipid and Protein Oxidation in Fish.” Aquaculture Research 50: 1393–1403.

[fsn370378-bib-0029] Hu, S. , Y. Luo , J. Cui , et al. 2013. “Effect of Silver Carp (*Hypophthalmichthys molitrix*) Muscle Hydrolysates and Fish Skin Hydrolysates on the Quality of Common Carp (*Cyprinus carpio*) During 4°C Storage.” International Journal of Food Science and Technology 48: 187–194. 10.1111/j.1365-2621.2012.03176.x.

[fsn370378-bib-0030] Ijaola, A. O. , D. O. Akamo , T. T. George , A. Sengul , M. Y. Adediji , and E. Asmatulu . 2024. “Algae as a Potential Source of Protein: A Review on Cultivation, Harvesting, Extraction, and Applications.” Algal Research 77: 103329.

[fsn370378-bib-0090] Iran Fisheries Organization . 2022. Statistical Year Book. Iran Fisheries Organization.

[fsn370378-bib-0031] Jasour, M. S. , and E. Z. Rahimabadi . 2011. “Effects of Refrigerated Storage on Fillet Lipid Quality of Rainbow Trout ( *Oncorhynchus mykiss* ) Supplemented by α‐Tocopheryl Acetate Through Diet and Direct Addition After Slaughtering.” Journal of Food Processing & Technology 2: 2. 10.4172/2157-7110.1000124.

[fsn370378-bib-0032] Javadian, S. R. , S. R. Shahosseini , and P. Ariaii . 2017. “The Effects of Liposomal Encapsulated Thyme Extract on the Quality of Fish Mince and *Escherichia coli* O157:H7 Inhibition During Refrigerated Storage.” Journal of Aquatic Food Product Technology 26, no. 1: 115–123. 10.1080/10498850.2015.1101629.

[fsn370378-bib-0083] Kang, H. J. , S. J. Kim , Y. S. You , M. Lacroix , and J. Han . 2013. “Inhibitory Effect of Soy Protein Coating Formulations on Walnut (Juglans regia L.) Kernels against Lipid Oxidation.” LWT‐Food Science and Technology 51, no. 1: 393–396.

[fsn370378-bib-0081] Khanzadi, S. , M. Hashemi , and M. Azizzadeh . 2021. “Antimicrobial Effect of Gel‐Type Nanoemulsion of Chitosan Coating Containing Essential Oils of Zataria multiflora and Bunium persicum on Pseudomonas Artificially Inoculated onto Salmon Fillets.” Medical Laboratory Journal 15, no. 3: 13–20.

[fsn370378-bib-0033] Ktari, N. , N. Fakhfakh , R. Balti , H. Ben Khaled , M. Nasri , and A. Bougatef . 2013. “Effect of Degree of Hydrolysis and Protease Type on the Antioxidant Activity of Protein Hydrolysates From Cuttlefish (*Sepia officinalis*) by‐Products.” Journal of Aquatic Food Product Technology 22: 436–448. 10.1080/10498850.2012.658961.

[fsn370378-bib-0034] Kumar, K. S. , J. Bindu , K. Elavarasan , A. K. Balange , and L. Narasimhamurthy . 2023. “Preservative Effect of Ready to Disperse Bioactive Edible Coating Powder From Fish Protein Hydrolysate Incorporated With Chitosan and Active Clove Oil on Tuna Fillets ( *Thunnus albacares* ) During Chilled Storage.” Indian Journal of Fisheries 70: 101–108. 10.21077/ijf.2023.70.2.132322-13.

[fsn370378-bib-0035] Li, P. , Q. Zhou , Y. Chu , W. Lan , J. Mei , and J. Xie . 2020. “Effects of Chitosan and Sodium Alginate Active Coatings Containing ε‐Polysine on Qualities of Cultured Pufferfish (*Takifugu obscurus*) During Cold Storage Peiyun.” International Journal of Biological Macromolecules 160: 418–428. 32422259 10.1016/j.ijbiomac.2020.05.092

[fsn370378-bib-0036] Lowry, O. H. , N. J. Rosebrough , A. L. Farr , et al. 1951. “Protein Measurement With the Folin Phenol Reagent.” Journal of Biological Chemistry 193: 265–275. 10.1016/0304-3894(92)87011-4. 14907713

[fsn370378-bib-0037] Lynch, M. P. , and C. Faustman . 2000. “Effect of Aldehyde Lipid Oxidation Products on Myoglobin.” Journal of Agricultural and Food Chemistry 48: 600–604. 10725121 10.1021/jf990732e

[fsn370378-bib-0038] Mahto, R. , S. Ghosh , M. K. Das , and M. Das . 2015. “Effect of Gamma Irradiation and Frozen Storage on the Quality of Fresh Water Prawn (*Macrobrachium rosenbergii*) and Tiger Prawn (*Penaeus monodon*).” LWT Food Science and Technology 61: 573–582.

[fsn370378-bib-0039] Mehdizadeh, T. , H. Tajik , A. Mojaddar , et al. 2020. “Chitosan‐Starch Film Containing Pomegranate Peel Extract and Thymus Kotschyanus Essential Oil Can Prolong the Shelf Life of Beef.” Meat Science 163: 108073. 10.1016/j.meatsci.2020.108073. 32014807

[fsn370378-bib-0040] Mirzapour‐Kouhdasht, A. , and M. Moosavi‐Nasab . 2020. “Shelf‐Life Extension of Whole Shrimp Using an Active Coating Containing Fish Skin Gelatin Hydrolysates Produced by a Natural Protease.” Food Science & Nutrition 8: 214–223. 10.1002/fsn3.1293. 31993147 PMC6977469

[fsn370378-bib-0041] Mohammadi, M. , A. Mirza Alizadeh , and N. Mollakhalili Meybodi . 2021. “Off‐Flavors in Fish: A Review of Potential Development Mechanisms, Identification and Prevention Methods.” Journal of Human, Environment, and Health Promotion 7: 120–128.

[fsn370378-bib-0042] Mondal, K. , V. V. Goud , and V. Katiyar . 2022. “Effect of Waste Green Algal Biomass Extract Incorporated Chitosan‐Based Edible Coating on the Shelf Life and Quality Attributes of Tomato.” ACS Food Science & Technology 2: 1151–1165.

[fsn370378-bib-0043] Mousaie, M. , M. Khodadadi , and M. Tadayoni . 2022. “Hydrolysate Protein From Brown Macroalgae (*Sargassum ilicifolium*): Antioxidant, Antitumor, Antibacterial, and ACE Inhibitory Activities.” Journal of Food Processing and Preservation 46: 1–10. 10.1111/jfpp.17020.

[fsn370378-bib-0044] Muñoz‐Tebar, N. , J. A. Pérez‐Álvarez , J. Fernández‐López , and M. Viuda‐Martos . 2023. “Chitosan Edible Films and Coatings With Added Bioactive Compounds: Antibacterial and Antioxidant Properties and Their Application to Food Products: A Review.” Polymers (Basel) 15: 396. 36679276 10.3390/polym15020396 PMC9864592

[fsn370378-bib-0045] Naghdi, S. , J. M. Lorenzo , R. Mirnejad , M. Ahmadvand , and M. Moosazadeh Moghaddam . 2023. “Bioactivity Evaluation of Peptide Fractions From Bighead Carp ( *Hypophthalmichthys nobilis* ) Using Alcalase and Hydrolytic Enzymes Extracted From Oncorhynchus Mykiss and Their Potential to Develop the Edible Coats.” Food and Bioprocess Technology 16: 1128–1148. 10.1007/s11947-022-02986-y.

[fsn370378-bib-0046] Naghdi, S. , M. Rezaei , and M. Abdollahi . 2021. “A Starch‐Based pH‐Sensing and Ammonia Detector Film Containing Betacyanin of Paperflower for Application in Intelligent Packaging of Fish.” International Journal of Biological Macromolecules 191: 161–170. 10.1016/j.ijbiomac.2021.09.045. 34536478

[fsn370378-bib-0048] Naghdi, S. , M. Rezaei , M. G. Heidari , R. Tahergorabi , J. M. Lorenzo , and F. Mirzaei . 2024. “Insights Into Fishery By‐Product Application in Aquatic Feed and Food: A Review.” Aquaculture International 32: 5851–5910. 10.1007/s10499-024-01447-x.

[fsn370378-bib-0049] Naghdi, S. , M. Rezaei , M. Tabarsa , and M. Abdollahi . 2023. “Fish Protein Hydrolysate From Sulfated Polysaccharides Extraction Residue of Tuna Processing by‐Products With Bioactive and Functional Properties.” Global Challenges 7: 2200214. 10.1002/gch2.202200214. 37020628 PMC10069310

[fsn370378-bib-0047] Naghdi, S. , M. Rezaei , N. Bahramifar , and B. Kuswandi . 2022. “Preparation and Characterization of Intelligent Color‐Changing Nanosensor Based on Bromophenol Blue and GONH_2_ Nanosheet for Freshness Evaluation of Minced Caspian Sprat ( *Clupeonella cultriventris* Caspia) Stored at 4°C.” Chemical Papers 76: 3133–3146. 10.1007/s11696-022-02095-2.

[fsn370378-bib-0082] Navarro‐Garcıa, G. , R. Pacheco‐Aguilar , L. Bringas‐Alvarado , and J. Ortega‐Garcıa . 2004. “Characterization of the Lipid Composition and Natural Antioxidants in the Liver Oil of Dasyatis Brevis and Gymnura Marmorata rays.” Food Chemistry 87, no. 1: 89–96.

[fsn370378-bib-0050] Ojagh, S. M. , M. Rezaei , S. H. Razavi , and S. M. H. Hosseini . 2010. “Effect of Chitosan Coatings Enriched With Cinnamon Oil on the Quality of Refrigerated Rainbow Trout.” Food Chemistry 120: 193–198. 10.1016/j.foodchem.2009.10.006.

[fsn370378-bib-0051] Onuh, J. O. , A. T. Girgih , R. E. Aluko , and M. Aliani . 2014. “In Vitro Antioxidant Properties of Chicken Skin Enzymatic Protein Hydrolysates and Membrane Fractions.” Food Chemistry 150: 366–373. 10.1016/j.foodchem.2013.10.107. 24360464

[fsn370378-bib-0052] Orak, H. H. , and S. Kayışoğlu . 2008. “Quality Changes in Whole, Gutted and Filleted Three Fish Species (*Gadus euxinus*, *Mugil cephalus*, *Engraulis encrasicholus*) at Frozen Storage Period (−26°C).” Acta Scientiarum Polonorum Technologia Alimentaria 7: 15–28.

[fsn370378-bib-0053] Papuc, C. , G. V. Goran , C. N. Predescu , and V. Nicorescu . 2017. “Mechanisms of Oxidative Processes in Meat and Toxicity Induced by Postprandial Degradation Products: A Review.” Comprehensive Reviews in Food Science and Food Safety 16: 96–123. 33371549 10.1111/1541-4337.12241

[fsn370378-bib-0054] Parvathy, U. , K. M. Nizam , A. A. Zynudheen , G. Ninan , S. K. Panda , and C. N. Ravishankar . 2018. “Characterization of Fish Protein Hydrolysate From Red Meat of *Euthynnus affinis* and Its Application as an Antioxidant in Iced Sardine.” Journal of Scientific and Industrial Research 77: 111–119.

[fsn370378-bib-0055] Pekkoh, J. , K. Ruangrit , C. Pumas , et al. 2021. “Transforming Microalgal Chlorella Biomass Into Cosmetically and Nutraceutically Protein Hydrolysates Using High‐Efficiency Enzymatic Hydrolysis Approach.” Biomass Conversion and Biorefinery 13: 6299–6315. 10.1007/s13399-021-01622-7.

[fsn370378-bib-0056] Pezeshk, S. , S. M. Ojagh , M. Rezaei , and B. Shabanpour . 2019. “Fractionation of Protein Hydrolysates of Fish Waste Using Membrane Ultrafiltration: Investigation of Antibacterial and Antioxidant Activities.” Probiotics and Antimicrobial Proteins 11: 1015–1022. 10.1007/s12602-018-9483-y. 30415461

[fsn370378-bib-0057] Piedrahíta Márquez, D. G. , C. A. Fuenmayor , and H. Suarez Mahecha . 2019. “Effect of Chitosan‐Propolis Edible Coatings on Stability of Refrigerated Cachama ( *Piaractus brachypomus* ) Vacuum‐Packed Fish Fillets.” Packaging Technology and Science 32: 143–153. 10.1002/pts.2422.

[fsn370378-bib-0058] Qara, S. , and M. B. Habibi . 2018. “Bioactive Properties of Kilka (*Clupeonella cultriventris* Caspi) Fish Protein Hydrolysates.” Journal of Food Measurement and Characterization 12: 2263–2270. 10.1007/s11694-018-9843-z.

[fsn370378-bib-0059] Sadeghi, S. , H. Jalili , S. O. Ranaei Siadat , and M. Sedighi . 2018. “Anticancer and Antibacterial Properties in Peptide Fractions From Hydrolyzed Spirulina Protein.” Journal of Agricultural Science and Technology 20: 673–683.

[fsn370378-bib-0060] Sáez, M. I. , M. D. Suárez , F. J. Alarcón , and T. F. Martínez . 2021. “Assessing the Potential of Algae Extracts for Extending the Shelf Life of Rainbow Trout ( *Oncorhynchus mykiss* ) Fillets.” Food 10: 910. 10.3390/foods10050910. PMC8143106 33919226

[fsn370378-bib-0061] Safari, R. , S. R. Shahhoseini , and S. R. Javadian . 2018. “Antibacterial and Antioxidant Effects of the Echinophora Cinerea Extract on Bighead Carp (*Aristichthys nobilis*) Fillet During Two Storage Conditions.” Journal of Applied Ichthyology 3: 13–24.

[fsn370378-bib-0062] Secci, G. , and G. Parisi . 2016. “From Farm to Fork: Lipid Oxidation in Fish Products. A Review.” Italian Journal of Animal Science 15: 124–136.

[fsn370378-bib-0063] Senadheera, T. R. L. , D. Dave , and F. Shahidi . 2021. “Antioxidant Potential and Physicochemical Properties of Protein Hydrolysates From Body Parts of North Atlantic Sea Cucumber (*Cucumaria frondosa*).” Food Production, Processing and Nutrition 3: 1–22. 10.1186/s43014-020-00049-3.

[fsn370378-bib-0064] Shafiei, R. , and T. Mostaghim . 2022. “Improving Shelf Life of Calf Fillet in Refrigerated Storage Using Edible Coating Based on Chitosan/Natamycin Containing Spirulina Platensis and *Chlorella vulgaris* Microalgae.” Journal of Food Measurement and Characterization 16: 145–161.

[fsn370378-bib-0065] Shah Hosseini, S. R. 2023. “Evaluation of Physical, Mechanical and Antimicrobial Properties of Pullulan Films Enriched With Free and Encapsulated Tragopogon Graminifolius DC. Extract for Use in Food Packaging.” Journal of Innovative Food Science & Technology 15: 133–146.

[fsn370378-bib-0066] Shah Hosseini, S. R. , R. Safari , and S. R. Javadiyan . 2021. “Evaluation Antioxidant Effects of Pullulan Edible Coating With Watercress Extract (*Nasturtiumn officinale*) on the Chemical Corruption of Fresh Beluga Sturgeon Fillet During Storage in a Refrigerator.” Iranian Scientific Fisheries Journal 30: 133–146.

[fsn370378-bib-0067] Shaibani, M. E. , B. Heidari , S. Khodabandeh , S. Shahangian , S. Mirdamadi , and M. Mirzaei . 2020. “Antioxidant and Antibacterial Properties of Protein Hydrolysate From Rocky Shore Crab, Grapsus Albolineathus, as Affected by Progress of Hydrolysis.” International Journal of Aquatic Biology 8: 184–193.

[fsn370378-bib-0068] Soltani, Z. , S. Naghdi , H. Sattar , et al. 2023. “Exploring the Antioxidant and Antibacterial Capacities of Padina Australis Extracts, and Their Utilization in Starch‐Based Coatings for Preserving Rainbow Trout (*Oncorhynchus mykiss*) Fillets.” Algal Research 74: 103234. 10.1016/j.algal.2023.103234.

[fsn370378-bib-0069] Song, H. Y. , Y. J. Shin , and K. B. Song . 2012. “Preparation of a Barley Bran Protein–Gelatin Composite Film Containing Grapefruit Seed Extract and Its Application in Salmon Packaging.” Journal of Food Engineering 113: 541–547.

[fsn370378-bib-0070] Srivastava, N. , K. Saurav , V. Mohanasrinivasan , K. Kannabiran , and M. Singh . 2010. “Antibacterial Potential of Macroalgae Collected From the Madappam Coast, India.” British Journal of Pharmacology and Toxicology 1: 72–76.

[fsn370378-bib-0071] Taghizadeh Andevari, G. , M. Rezaei , M. Tabarsa , and T. Rustad . 2019. “Extraction, Partial Purification and Characterization of Alkaline Protease From Rainbow Trout ( *Oncorhynchus mykiss* ) Viscera.” Aquaculture 500: 458–463. 10.1016/j.aquaculture.2018.10.052.

[fsn370378-bib-0072] Tsegay, Z. T. , S. Agriopoulou , M. Chaari , S. Smaoui , and T. Varzakas . 2024. “Statistical Tools to Optimize the Recovery of Bioactive Compounds From Marine Byproducts.” Marine Drugs 22: 1–34. 10.3390/md22040182. PMC11050780 38667799

[fsn370378-bib-0073] Ucak, I. , R. Khalily , C. Carrillo , I. Tomasevic , and F. J. Barba . 2020. “Potential of Propolis Extract as a Natural Antioxidant and Antimicrobial in Gelatin Films Applied to Rainbow Trout ( *Oncorhynchus mykiss* ) Fillets.” Food 9: 1584. 10.3390/foods9111584. PMC7693740 33139596

[fsn370378-bib-0074] Uçak, I. , Y. Özogul , and M. Durmuş . 2011. “The Effects of Rosemary Extract Combination With Vacuum Packing on the Quality Changes of Atlantic Mackerel Fish Burgers.” International Journal of Food Science and Technology 46: 1157–1163. 10.1111/j.1365-2621.2011.02610.x.

[fsn370378-bib-0075] Wald, M. , K. Schwarz , H. Rehbein , B. Bußmann , and C. Beermann . 2016. “Detection of Antibacterial Activity of an Enzymatic Hydrolysate Generated by Processing Rainbow Trout By‐Products With Trout Pepsin.” Food Chemistry 205: 221–228. 10.1016/j.foodchem.2016.03.002. 27006234

[fsn370378-bib-0076] Weng, W. Y. , K. Osako , and M. Tanaka . 2009. “Oxygen Permeability and Antioxidative Properties of Edible Surimi Films.” Fisheries Science 75: 233–240. 10.1007/s12562-008-0024-6.

[fsn370378-bib-0077] Xie, X. , X. Zhai , M. Chen , et al. 2023. “Effects of Frozen Storage on Texture, Chemical Quality Indices and Sensory Properties of Crisp Nile Tilapia Fillets.” Aquaculture and Fisheries 8: 626–633.

[fsn370378-bib-0078] Yıldırım‐Yalçın, M. , H. Sadıkoğlu , and M. Şeker . 2021. “Optimization of Mechanical and Antioxidant Properties of Edible Film Based on Grape Juice and Cross‐Linked Maize Starch and Evaluation of Its Effects on the Storage Quality of Fresh‐Cut Pineapple.” Journal of Food Measurement and Characterization 15: 4669–4678. 10.1007/s11694-021-01038-x.

[fsn370378-bib-0079] Yu, J. , Y. Hu , M. Xue , et al. 2016. “Purification and Identification of Antioxidant Peptides From Enzymatic Hydrolysate of Spirulina Platensis.” Journal of Microbiology and Biotechnology 26: 1216–1223. 10.4014/jmb.1601.01033. 27090190

